# Evaluating the In Vitro Digestibility of Black Soldier Fly (*Hermetia illucens* L.) Larvae as Potential Feed Ingredient in Dairy Cattle Silage‐Based Diets: A Focus on Proteins

**DOI:** 10.1111/jpn.70098

**Published:** 2026-07-30

**Authors:** Maria Giulia Bonomini, Marica Simoni, Matteo Profeti, Federico Righi, Augusta Caligiani

**Affiliations:** ^1^ Department of Food and Drug University of Parma Parma Italy; ^2^ Department of Veterinary Sciences University of Parma Parma Italy

**Keywords:** alternative protein, black soldier fly (BSF), dairy cattle nutrition, in vitro digestion, insect digestibility, rumen fermentation

## Abstract

Dietary protein has a substantial economic impact on cattle feed, and it is also a critical component for animal growth and productivity. Several environmental issues related to soybeans have led to an increased interest in novel protein sources, such as insects. However, research on the inclusion of insects in bovine diets is limited, also due to the current European legislation on the topic. For this reason, this study aimed at investigating the use of black soldier fly (BSF) larvae as a soybean replacer in dairy cattle diet. Three iso‐nitrogenous and iso‐energetic diets for lactating dairy cattle were formulated, including (i) soybean, (ii) a commercial protein‐rich feedstuff and (iii) BSF larvae as a main protein source. After an in vitro bovine digestion, dry matter digestibility (DMD) and crude protein digestibility (CPD), were determined. Additionally, for the first time to our knowledge, individual amino acids and chitin digestibility, degree of hydrolysis (DH%), molecular weight distribution of proteins via SDS‐PAGE gel electrophoresis and HR‐MS analysis of peptides released after digestion were also assessed. The results showed that the formulated diets did not differ in terms of DMD, CPD, amino acid solubilization and DH%, highlighting the potential of replacing soybean meal with insects without negatively affecting these parameters. In addition, 50% of chitin was solubilized at the end of the intestinal phase, both in individually digested BSF larvae and in the diet formulated with BSF larvae, meaning that chitinolytic microorganisms were present in the rumen and that chitin represents a potential source of carbon and nitrogen for ruminants.

## 1 Introduction

The food sector accounts for one‐quarter of the overall greenhouse gas emissions worldwide, with cattle for dairy production being the third most contributing per kg of food products (Ritchie et al. 2022). As, at present, the general demand for animal‐based products is increasing (for instance, since 1994, the global production of cattle meat has increased from 51.0 to 69.5 million tons/year, and that of cheese from whole cow milk from 11.2 to 22.6 million tons/year) (FAO 2025), effective dietary management is becoming of the utmost importance to reduce the environmental impact of this sector (Ritchie et al. 2022; Simoni et al. 2024; Sogari et al. 2023). Among ruminant feed nutrients, dietary protein not only has a substantial economic impact on diet costs, but it is also a critical component for animal growth and productivity (Hristov et al. 2019). Studies have shown that the amino acid (AAs) profile of dairy cows' feed is strictly related to milk protein content; however, if exceeding the animal's dietary requirements or if not efficiently utilized, the excess nitrogen derived from protein degradation is excreted by urine and faeces and actively contributes to increasing the environmental impact (Estes et al. 2022; Hristov et al. 2019; Paz et al. 2014). Therefore, improving the efficiency of nitrogen metabolism in dairy cattle would help in decreasing feed costs and the environmental impact of the sector, while maintaining high‐quality standards of production.

In ruminants, a part of dietary protein (rumen degradable protein [RDP]) is necessary to support microbial growth in the rumen (Estes et al. 2022). The proportion of dietary protein which is not degradable by ruminal microorganisms (rumen undegradable protein [RUP]) escapes the rumen and is potentially digested in the abomasum and small intestine. Thus, the balance between RDP and RUP is fundamental for the optimization of protein requirements in ruminants, and fast, precise methods for assessing these parameters are required (Estes et al. 2022; Hristov et al. 2019; Zain et al. 2024). Several protocols for the determination of RDP or RUP are available in literature: among these, in vitro methods constitute a valuable alternative to in vivo studies as they are more cost‐ and time‐effective. Ross et al. (2013) developed a three‐step procedure consisting of a ruminal phase, abomasal phase and intestinal phase of 16, 1 and 24 h, respectively, using rumen fluid and a mixture of different enzymes to mimic the in vivo conditions of bovine digestion. Although the protocol has not been published yet in a peer‐reviewed journal, it is already in use in commercial laboratories for the determination of intestinal protein digestibility of feeds and rumen‐protected ingredients bioavailability (Estes et al. 2022).

While soybean meal, the most used protein source in feed globally, is largely a by‐product of the vegetable oil industry, its volume of consumption presents several issues from an environmental point of view, leading to a worrying increase in deforestation and loss of biodiversity (Spranghers et al. 2018). For these reasons, interest in novel protein sources for feed has increased in recent years. Among these, insects represent a valuable substitute: in fact, their AAs profile is comparable to the soybean meal one, and they can be reared on a variety of organic by‐products, thus having low maintenance costs and aiding to contribute to a future system of circular economy (Ismail et al. 2020; Spranghers et al. 2018). Among insect species, *Hermetia illucens* or black soldier fly (BSF) is one of the most promising species, as it represents a source of high‐quality proteins, needs few resources to be reared and has a short reproductive cycle, allowing to obtain high nutritional value biomass in a short time (Hawkey et al. 2021; Lu et al. 2022; van Huis and Gasco 2023). In addition, BSF larvae and their fractions have well‐established bioactive properties, such as antibacterial and prebiotic activities (Auza et al. 2020; Choi et al. 2012; Dong et al. 2021; Harlystiarini et al. 2019; Refael et al. 2022; Van Moll et al. 2022), further highlighting their potential to be used as a feed ingredient (Nekrasov et al. 2022). Crucially, insect components such as chitin and its derivatives can exert positive biological effects on ruminant species, acting in the modulation of microbial populations, improving fermentation efficiency, having immune‐modulatory and antimicrobial effect and overall supporting animal production performance without compromising total tract digestibility (Shah et al. 2022). These properties are frequently associated with neutral ruminal fermentation dynamics, maintaining optimal volatile fatty acid profiles and ammonia nitrogen levels (Lu et al. 2024; Maggitt et al. 2025).

However, despite the promising results in the literature, the European legislation (Regulation [EC] No 999/2001) highlights a major obstacle in the application of insects for ruminants, which is the potential risk of bovine spongiform encephalopathy transmission, and only a few studies (Renna et al. 2023) have attempted to provide information on the matter, limiting the policymakers in thoroughly evaluating this potential hazard. However, the use of insects as feed in ruminant diets is still of great interest, as they represent a promising alternative to conventional protein sources, and due to this, scientific research on this topic has increased in the past years (Renna et al. 2023; Toral et al. 2022). Nonetheless, explicit data tracing the metabolic fate of insect nutrients across the ruminal gastrointestinal tract remains scarce. In particular, comprehensive evaluations of how the complex insect exoskeleton matrix alters site‐specific protein degradation, or how chitin interacts with the true AAs availability under multi‐phase digestion, are currently lacking (Ahmed et al. 2021; Fukuda et al. 2022; Jayanegara et al. 2017; Shah et al. 2022).

Based on these premises, this study aimed at investigating in vitro the use of *H. illucens* whole larvae as protein source in lactating cow's diet as substitute for soybean meal, focusing on common feed quality parameters such as dry matter digestibility (DMD) and crude protein digestibility (CPD), but also, for the first time to the best of our knowledge, on individual AAs and chitin digestibility (ChitD), degree of hydrolysis of proteins (DH%), Sodium Dodecyl Sulphate‐Polyacrylamide Gel Electrophoresis (SDS‐PAGE) gel electrophoresis at ruminal and intestinal digestion endpoints and HR‐MS analysis of peptides released after digestion.

## 2 Materials and Methods

### 2.1 Samples and Diet Formulation

The *H. illucens* L. larvae were purchased from the company Société APPI (Pont St Martin, France). All the other dietary ingredients were collected in local farms, characterized for their nutrient components, and their composition was inputted in the Nutritional Dynamic System (NDS) Professional dynamic formulation software version 6.55 (RUM&N, Reggio Emilia, Emilia‐Romagna, Italy). The latter was employed to formulate three iso‐energetic and iso‐nitrogenous diets (Table 1) based on the nutritional recommendation for Holstein dairy cows (NASEM [National Academies of Sciences, Engineering, and Medicine] 2021). All diets contained a standard protein feedstuff complying with the regulations for production of both Grana Padano PDO (Reg. EU No 584/2011) and Parmigiano Reggiano PDO (European Parliament 2024/1143). The control diet (D‐S) was a typical total mixed ration (TMR) of the Grana Padano area including soybean meal, protein feedstuff and hydrogenated fat; soybean meal and hydrogenated fat were totally replaced by protein feedstuff in the second diet (D‐N) and by BSF larvae in the third diet (D‐BSF). The diets were ground with a Retsch SK mill (Bauuknecht, Stuttgart, Germany) to pass a 1 mm mesh screen.

**Table 1:** Feed ingredients and chemical composition of the experimental total mixed rations (D‐S: soybean diet; D‐N: protein feedstuff mix diet; D‐BSF: black soldier fly larvae diet).

	D‐S	D‐N	D‐BSF
Diets (% DM)			
Corn silage	39.6	39.4	39.4
Alfalfa silage	8.8	8.8	8.8
Alfalfa hay	8.8	8.8	8.8
Corn meal	8.8	8.8	8.8
Wheat bran	4.4	4.4	4.4
Beet pulp	8.8	8.8	8.8
Soybean meal	4.2	—	—
Protein feedstuff mix^a^	15.3	20.6	15.3
BSF larvae^b^	—	—	5.3
Sodium bicarbonate	0.6	0.6	0.6
Hydrogenated fat	0.9	—	—
Mcal/kg ration	6.9	6.7	6.9
Chemical composition (% DM)			
Dry matter	87.9	88.0	88.0
Crude protein	16.2 ± 0.02	16.0 ± 0.04	16.2 ± 0.15
Ether extract	3.5 ± 0.5	3.1 ± 0.2	5.2 ± 0.1
Starch	1.8	1.9	1.9
Ash	8.0 ± 0.4	8.1 ± 0.1	7.7 ± 0.1
aNDF	41.0 ± 4.95	38.1 ± 1.76	36.8 ± 0.52
ADF	22.7 ± 2.18	22.5 ± 3.79	21.5 ± 0.83
ADL	3.6 ± 1.00	3.3 ± 0.95	3.7 ± 1.15
Chitin	*/*	*/*	0.55 ± 0.04

^a^ Composition (% DM): Soybean meal 37.7%, soybean hulls 12.8%, corn gluten feed 8.4%, whole soybean roasted 3.2%, linseeds cake 7.7%, sunflower meal 4.4%, Barley meal 4.3%, sodium bicarbonate 4.6%, corn gluten meal 4%, potato protein concentrate 3.5%, hydrogenated fat 2.6%, calcium carbonate 2%, calcium monophosphate 1.3%, cane molasses 0.8%, sodium chloride 1%, magnesium oxide 0.3%, mineral‐vitamin mix 1%.

^b^ Chemical composition (% DM): Dry matter, % as fed 94.4%, crude protein 31.6%, ether extract 41.8%, ash 5.0%, chitin 10.5%.

### 2.2 Chemical Composition Analyses

The chemical composition of the three diets and of the whole BSF larvae was determined following standard methods for feed analyses (European Commission [EC] 2009). Briefly, dry matter (DM) content was determined after oven‐drying at 103°C overnight. Ash content was determined gravimetrically after incineration at 550°C for 4 h. The ether extract was determined by the Soxhlet method. Fibre fractions (hemicellulose, cellulose and lignin) were determined according to Mertens et al. (2002) and Robertson and Van Soest (1981) and as described by Simoni et al. (2024). In brief, the amylase‐treated neutral detergent fibre without residual ash (aNDF_om_) was determined with the use of heat‐stable amylase, without sodium sulfite and adjusted for their ash content. Fibre fractions analysis was performed sequentially to determine the difference between hemicellulose and cellulose. Nitrogen content was determined by combustion digestion of the sample at 900°C in an excess of oxygen by Dumatherm (Gerhardt GmbH &Co, Königswinter, Germany) as described by Mihaljev et al. (2015). The protein content was calculated by multiplying the value obtained by the Dumas method by a standard nitrogen‐to‐protein conversion factor for the three formulated diets (6.25), and by a conversion factor that only considers nitrogen from protein sources for the whole BSF larvae (4.76), thus excluding chitin‐derived nitrogen (Janssen et al. 2017). Chitin content was determined by UPLC‐ESI/MS analysis for the whole BSF larvae and for the insect‐enriched diet as described by Bonomini et al. (2024).

### 2.3 Bovine Digestibility Assay

Bovine digestibility was determined using an in vitro technique for the three diets (D‐S, D‐N and D‐BSF) and for whole BSF larvae. The digestion trial was carried out in triplicate for both the ruminal and abomasal/intestinal phases. At the end of each digestion phase, the DMD and CPD of the samples were determined.

*Ruminal fermentation*: Ruminal digestibility was assessed in an in vitro batch system using a pooled rumen fluid collected at the slaughterhouse from four cows. The rumen fluid was kept in an anaerobic condition at 39°C, blended and filtered through four layers of cheesecloth. The filtered rumen fluid was inoculated at a ratio of 1:4 with Van Soest buffer solution in each flask containing 0.5 g of diet or whole BSF larvae. Flasks were incubated in an in vitro batch fermentation system (Goering and Van Soest 1970). A blank sample consisting only of Van Soest buffer and rumen fluid was also included for each phase. The flasks were incubated at 39°C for 16 h under anaerobic conditions. The flasks allocated to ruminal digestion were then removed from the water bath and refrigerated for further analyses, while those allocated to intestinal digestion were kept in the fermentation system at the same temperature to complete the subsequent digestion phase.

*Abomasal and intestinal digestion*: After the ruminal phase, the abomasal and intestinal phases were performed following the procedure described by Ross et al. (2013), with a few modifications. The ruminal digesta were acidified to pH 2 with HCl 3 M, subsequently added with 2 mL of a pepsin solution (70 U/mL) in HCl 0.013 M, and the flasks were capped and incubated for 1 h at 39°C. After the abomasal phase, the pH was adjusted to 5 with NaOH 2 M, and 10 mL of a mixture of enzymes in phosphate buffer 1.8 M (trypsin, chymotrypsin, α‐amylase, bile and lipase) were added. The flasks were capped and incubated at 39°C for 24 h.

The ruminal and intestinal digesta were filtered with borosilicate crucibles (Robu Glass Filter–ROBU H3, Borosilicate 3.3, 30 mL—Por. 2, Hattert, Germany) added with 0.4 g of celite to facilitate the filtration phase. The filtered digesta solutions were collected in Falcon tubes and stored at −20°C for further analysis, while the solid undigested residues in the crucibles were rinsed three times with 39°C distilled water and dried overnight at 103°C for DMD determination. The solid undigested residue in the crucible was then collected and analysed for the N content by the Dumas method as described by Simoni et al. (2024) to determine the CPD.

#### 2.3.1 DH% of Proteins in Solution: OPA/NAC Analysis

The DH% was determined for all the filtered digesta solutions following the procedure described by Bonomini et al. (2024). Briefly, the filtered digesta solutions obtained after each endpoint of digestion (ruminal and intestinal) were filtered with 0.45 µm syringe filters. An aliquot of 20 µL of each solution was added to 2.4 mL of OPA/N‐acetyl cysteine (NAC) reagent (OPA 50 mM, NAC 50 mM, SDS 2%, borate buffer 0.1 M at pH 9.5) and incubated in darkness for 10 min. Then, the absorbance of the free N groups at 340 nm was measured using the Agilent Cary 60 UV‐Vis spectrophotometer. Sample intrinsic absorbance was also measured by adding 20 µL of sample solution to 2.4 mL of distilled water, and the value obtained was subtracted from the absorbance with the OPA/NAC reagent. The DH% of proteins in solution was then calculated as the ratio between the free nitrogen groups and the total nitrogen groups of the protein in the filtrate solution. The latter was obtained indirectly as a difference between the total N in the diet and the N in the dried solid undigested residue, both analysed by the Dumas method. Finally, the average peptide length (APL) was also calculated as 100/DH.

### 2.4 Analysis of AAs and Chitin

Total AAs and chitin content were determined as described by Bonomini et al. (2024) on BSF whole larvae, diets (Supporting Information S1: Table S1) and dried solid undigested residues after ruminal and intestinal digestion endpoints. Then, the specific amino acid digestibility (AAD) and ChitD for each endpoint of digestion (ruminal or intestinal) were calculated as:

$$\begin{array}{lll} AAD\% & = & 100-\frac{g\;AA_{residue}-g\;AA_{blank}}{g\;AA_{sample}}\\ ChitD\% & = & 100-\frac{g\;CHIT_{residue}}{g\;CHIT_{sample}} \end{array}$$

Where g AA_residue_, g AA_blank_ or g CHIT_residue_ is the quantity (g) of amino acid or of chitin in the solid undigested residue, while g AA_sample_ or g CHIT_sample_ is the quantity (g) of the same amino acid or of chitin in the diet.

### 2.5 SDS‐PAGE

SDS‐PAGE analysis was carried out to study the molecular weight (MW) distribution of diets, BSF and filtered digesta solutions. Briefly, the protein fraction of the diets and BSF was extracted using a buffer solution (urea 4 M, ammonium bicarbonate 100 mM, dithiothreitol 5 mM) for 2 h at room temperature with the Multi Bio RS‐24 rotator, while the filtered digesta solutions were filtered with 0.45 µm syringe filters. Then, the pH of all samples was neutralized. The SDS‐PAGE analysis was performed with the 12% Criterion XT Bis/Tris gels (Bio‐Rad, Hercules, CA, USA) following the procedure described by Bonomini et al. (2024) and using 50 µg of protein for each sample. The gel was acquired with the Bio‐Rad GS‐800 Calibrated Imaging Densitometer.

### 2.6 UHPLC/IM‐QTOF‐MS Analysis of Peptides in the Filtered Digesta Solutions

The peptides released from the ruminal and intestinal digestions of the whole BSF larvae and of the D‐BSF were analysed by HR‐MS analysis with the VIon IMS QTof mass spectrometer (Waters, Milford, USA). Data processing was carried out with the UNIFI software (Waters, Milford, USA). The chromatographic, mass spectrometric and data processing parameters used were those reported by Accardo et al. (2022), while the accession numbers (UniProt database) used as a list of expected proteins in solution were those reported in a previous study on the same insect matrix (Bonomini et al. 2024), derived from an LC/HR‐MS untargeted analysis with the LTQ‐Orbitrap XL mass spectrometer. Data filtering was carried out following the parameters described in Bonomini et al. (2024).

### 2.7 Statistical Analysis

The software IBM SPSS Statistics version 28.0.1.1 (SPSS Inc., Chicago, IL, USA) was used for statistical analysis. The data were tested for normality and analysed with the one‐way analysis of variance (ANOVA) test followed by the Tukey *b* post hoc test. The level of significance was set at 0.05.

## 3 Results and Discussion

While acknowledging the general scientific principle that the digestibility of a single feed ingredient should not be directly compared to that of a formulated diet, we included a comparison of the single insect ingredient against the formulated diets (soy‐based, protein feedstuff mix‐based and insect‐based) to provide novel insights into the specific digestive fate of the insect components, specifically proteins and chitin, within the ruminant gut environment. This approach allows for a preliminary and comparative understanding of how the characteristics of the insect ingredient influence the overall diet digestibility, given the current scarcity of data on insect utilization in ruminants and the complexity of insect digestion due to the presence of chitin and chitin‐bound proteins.

### 3.1 DMD and CPD

To evaluate the suitability of feed ingredients for ruminants, it is of utmost importance to determine their DMD and CPD of both ruminal and intestinal phases. These values are a measure of feedstuff nutrient availability, as they represent the proportion of DM or CP, respectively, that is digested and can therefore be used by the animal. In addition, sufficient RDP and carbohydrates are required to support microbial growth in the rumen, while adequate RUP is needed to meet the animal's N requirement, thus reinforcing the importance of the CPD of diets for ruminants (Fukuda et al. 2022; Paz et al. 2014; Zain et al. 2024). Due to these reasons, DMD and CPD of ruminal and intestinal endpoints were determined for the tested diets (Table 2) after in vitro digestion. The DMD and CPD of individually digested BSF were also assessed and compared to other common protein‐rich feed ingredients (Table 3).

**Table 2:** Dry matter digestibility (DMD) and crude protein digestibility (CPD) of the three diets (D‐S: soybean diet; D‐N: protein feedstuff mix diet; D‐BSF: black soldier fly larvae diet) and of black soldier fly (BSF) whole larvae meal.

	Ruminal endpoint		Intestinal endpoint	
Diet	DMD (% DM)	CPD (% DM)	DMD (% DM)	CPD (% DM)
D‐S	59.2 ± 3.52^b^	68.3 ± 4.07^a^	71.1 ± 2.57^a^	93.5 ± 0.45^a^
D‐N	61.3 ± 0.74^b^	72.5 ± 1.40^a^	72.9 ± 3.99^a^	94.3 ± 1.88^a^
D‐BSF	57.8 ± 2.62^b^	71.6 ± 5.49^a^	71.4 ± 4.87^a^	94.4 ± 0.96^a^
BSF	26.8 ± 0.94^a^	62.6 ± 4.16^a^	66.9 ± 3.69^a^	93.0 ± 1.73^a^

*Note:* The values are the average of three replicates ± SD. Different letters within the same column refer to statistically significant differences between samples (one‐way ANOVA, Tukey's *b* post hoc test, *α* = 0.05).

**Table 3:** Dry matter digestibility (DMD) and crude protein digestibility (CPD) of black soldier fly (BSF) whole larvae meal compared to other protein‐rich feed ingredients.

	Ruminal endpoint		Intestinal endpoint	
Feed ingredient	DMD (% DM)	CPD (% DM)	DMD (% DM)	CPD (% DM)
BSF	26.8 ± 0.94	62.6 ± 4.16	66.9 ± 3.69	93.0 ± 1.73
SBM^a^	78.9	68.8	/	/
CM^a^	68.6	75.7	/	/
BM^a^	22.6–83.5	29.8–85.3	/	/
CC^b^	64.7	/	/	/
MOC^b^	66.2	/	/	/

*Note:* The values of BSF are the average of three replicates ± SD.

Abbreviations: BM = blood meal, CCM = cottonseed cake, CM = canola meal, MOC = mustard oil cake, SBM = soybean meal.

^a^ Paz et al. (2014).

^b^ Kumar et al. (2015).

Regarding experimental diets, these were diet formulas typically used in lactating dairy cows in northern Italy, and their chemical composition was in line with the ones reported in the literature for the same area (Simoni et al. 2024). No differences in DMD were observed for both the ruminal and intestinal endpoints. To the best of our knowledge, no data on the use of insects in dairy cattle nutrition are available in the literature. However, the values of ruminal DMD were in line with the findings of Fukuda et al. (2022), who studied the in vivo nutrient digestibility at ruminal level of diets formulated with BSF larvae as protein supplement in beef steers. The results are also in line with the in vitro DMD values estimated by Jayanegara et al. (2017), which ranged between 53.2% and 66.6%, and no differences were detected in this parameter when BSF was used as a partial substitution of napier grass or soybean meal.

No differences were found for CPD either for ruminal or intestinal endpoints. Although the diets were formulated to be iso‐energetic and iso‐nitrogenous, the D‐BSF was expected to have a slight reduction compared to the two other formulated diets in both DMD and CPD due to the presence of chitin, which has been reported to impair nutrient digestibility. This highlights that, while a standalone alternative ingredient may exhibit lower individual digestibility due to isolated matrix constraints, such as high lipid concentrations or intact chitin, its putative negative impacts are completely neutralized when integrated into a complex diet. As a matter of fact, the DMD and CPD of BSF as a single feed ingredient were statistically lower than those of the diets at the ruminal endpoint, as discussed in the following paragraph. However, the absence of differences suggests that the replacement of soybean meal in D‐S with the protein feedstuff or with BSF at the tested proportions, while maintaining iso‐energetic and iso‐nitrogenous properties, did not negatively affect the overall nutrient breakdown and the specific CPD in either ruminal or intestinal phases, resulting in negligible differences from a biological standpoint.

In fact, when comparing the DMD of the BSF after ruminal phase to other feed ingredients (Table 3), it resulted substantially lower, suggesting that the microbiome in the rumen was not fully able to exploit the larvae as growing substrate. This was probably due to the chitin content of the larvae (Table 1), commonly a limiting factor for livestock feed both in non‐ruminant and ruminant diets (Fukuda et al. 2022): this N‐acetylglucosamine polymer is an N‐containing polysaccharide and is the main constituent of the insect's exoskeleton. It is usually highly resistant to both acid and enzymatic digestion due to its compact structure, extensive hydrogen bonding and the lack of chitinase enzymes in most organisms (Churchward‐Venne et al. 2017; Pedrazzani et al. 2024). These results were in line with the literature, as it was noticed that ruminal fermentation of BSF larvae or their chitin fraction decreased the ruminal DMD of the diet (Jayanegara et al. 2017).

The lower DMD might also be due to the intrinsic high lipid and low digestible carbohydrate content of the BSF, and not caused by an antagonistic effect of insect components: in fact, despite the DMD of BSF as single ingredient was lower at ruminal phase compared to the DMD of the tested diets, the CPD of BSF was similar to the ones of the same diets, indicating that the proteinaceous nitrogen of the insect was utilized to the same extent as the nitrogen of the other protein‐rich feed ingredients, thus probably meaning that the insect component not fermented and solubilized in the rumen are lipids. Additionally, the DMD of the BSF as single ingredient at intestinal endpoint was matching the ones of the tested diets, meaning that the abomasal and intestinal digestion phases, since they involve the use of different enzymes and bile salts, were able to compensate the lower ruminal DMD of whole BSF larvae, and thus, theoretically, the protein raw materials, despite their different composition, supply overall a similar amount of nutrients compared to the three diets, highlighting the efficiency of ruminants' multi‐phase digestion in breaking down dietary organic components.

### 3.2 AA and Chitin Solubilization After Digestion

In animal nutrition, the CPD is not the only factor to consider when formulating a balanced diet. Diets with the same protein content can indeed exhibit different AA profiles and solubilization capacity, depending on the protein source of their ingredients. This is particularly relevant for D‐BSF, as the presence of chitin might hinder nutrient digestibility (Churchward‐Venne et al. 2017). However, chitin supplementation in ruminant diets might be beneficial in reducing methane (CH_4_) emission, thereby contributing to the mitigation of the dairy cattle sector's environmental impact (Ahmed et al. 2021). Therefore, AA and ChitD must be carefully evaluated to ensure an adequate supply of AAs and potentially modulate CH_4_ production. For this reason, the solubilization of individual AAs and of chitin after both digestion endpoints (ruminal and intestinal) was evaluated by UPLC‐ESI/MS analysis. The results on the proportion of solubilized AAs of the three experimental diets are reported in Table 4.

**Table 4:** Solubilized amino acids (% AA) after ruminal and intestinal digestion of the three diets (D‐S: soybean diet; D‐N: protein feedstuff mix diet; D‐BSF: black soldier fly larvae diet).

	Ruminal solubilization (% AA)			Intestinal solubilization (% AA)		
	D‐S	D‐N	D‐BSF	D‐S	D‐N	D‐BSF
ala	81.8 ± 0.76	81.1 ± 0.34	77.7 ± 5.11	95.5 ± 0.36	95.8 ± 0.45	96.0 ± 1.22
arg	65.8 ± 2.75	67.2 ± 3.03	61.8 ± 5.49	89.2 ± 2.53	93.4 ± 1.94	88.0 ± 1.36
asp	81.1 ± 1.18	79.0 ± 0.44	74.1 ± 5.83	97.6 ± 0.17	97.1 ± 0.03	97.7 ± 1.07
glu	82.1 ± 0.24	81.6 ± 0.34	78.2 ± 4.51	97.0 ± 0.28	97.1 ± 0.18	97.3 ± 1.17
gly	77.1 ± 1.59	78.2 ± 0.34	73.3 ± 5.11	93.2 ± 0.61	94.6 ± 1.09	93.5 ± 1.68
his	32.2 ± 2.87^a^	43.8 ± 0.27^a^	32.8 ± 7.25^a^	63.5 ± 6.89^a^	81.5 ± 4.46^b^	64.1 ± 1.86^a^
ile	73.8 ± 0.83	72.4 ± 1.89	65.9 ± 6.38	93.8 ± 0.60	94.9 ± 1.14	94.0 ± 1.78
leu	79.1 ± 0.84	78.9 ± 0.68	73.0 ± 5.20	94.4 ± 0.29	95.5 ± 1.11	94.4 ± 1.58
lys	19.1 ± 4.05	5.0 ± 4.19	12.9 ± 0.00	85.6 ± 2.02	86.8 ± 0.42	86.7 ± 1.81
phe	72.4 ± 2.01	74.9 ± 0.73	68.9 ± 4.75	90.5 ± 1.46	93.9 ± 1.62	89.8 ± 1.51
pro	87.4 ± 0.42	87.1 ± 0.21	84.4 ± 3.10	96.4 ± 0.18	96.8 ± 0.74	96.4 ± 0.96
ser	77.4 ± 1.54	78.6 ± 0.60	74.4 ± 4.95	93.9 ± 0.58	95.4 ± 1.00	94.2 ± 1.11
thr	73.1 ± 2.60	73.9 ± 0.48	69.0 ± 5.47	93.5 ± 0.75	94.9 ± 1.02	94.2 ± 1.66
tyr	65.6 ± 1.83	68.8 ± 0.64	64.6 ± 5.22	87.1 ± 2.26	92.8 ± 2.26	87.2 ± 1.18
val	77.0 ± 0.93	76.2 ± 1.05	70.4 ± 4.85	94.5 ± 0.47	95.3 ± 0.80	94.8 ± 1.59

*Note:* The values are the average of two replicates ± SD. Different letters within the same line and the same endpoint refer to statistically significant differences between samples (one‐way ANOVA, Tukey's *b* post hoc test, *α* = 0.05).

The percentage of solubilized AAs at ruminal and intestinal endpoints, except for His solubilization after the intestinal phase which was higher in D‐N, was equal for all AAs among the diets, in line with previous results on CPD, suggesting that the partial substitution of soybean meal by BSF does not influence the specific AA release negatively.

The AA solubilization of BSF, individually digested, was also assessed. Although the results (Table 5) were always lower than the three diets at ruminal endpoint (Table 4), consistently with the CPD results, after abomasal and intestinal phases, the values of solubilized AA of the BSF as individual ingredient were comparable to those of the three diets, accounting for about 90% of solubilization. This probably means that, despite being unable to cleave proteins at high percentages, previously to the low abomasal pH, the ruminal microorganisms might have uncovered several cleavage sites, thus allowing the gastric and intestinal enzymes to digest insect proteins to the same extent as the three tested diets.

**Table 5:** % Solubilized amino acids (% AA) after ruminal and intestinal digestion of black soldier fly (BSF) whole larvae meal.

	Ruminal solubilization (% AA)	Intestinal solubilization (% AA)
ala	46.5 ± 0.82	93.6 ± 1.32
arg	31.9 ± 0.76	89.5 ± 1.23
asp	47.2 ± 1.02	93.4 ± 1.96
glu	52.9 ± 0.07	94.0 ± 1.62
gly	42.4 ± 1.04	89.1 ± 1.92
his	3.2 ± 0.36	68.0 ± 1.55
ile	35.3 ± 1.15	89.9 ± 2.39
leu	45.3 ± 1.00	91.4 ± 2.11
lys	10.6 ± 1.14	90.8 ± 2.06
phe	42.0 ± 1.31	87.3 ± 2.37
pro	51.0 ± 0.02	94.2 ± 1.20
ser	41.3 ± 0.85	91.5 ± 2.05
thr	41.0 ± 0.22	91.3 ± 2.26
tyr	42.8 ± 0.81	89.9 ± 1.71
val	41.2 ± 0.31	91.7 ± 2.02

*Note:* The values are the average of two replicates ± SD.

However, the values of AA solubilization after ruminal phase are in stark contrast with the previously described data on CPD of BSF (Table 3), where, at ruminal endpoint, BSF had similar CPD to that of the three diets. This might be due to the fact that CPD was determined with the Dumas method; thus, nitrogen contribution from non‐protein nitrogen, true protein or from chitin was unrecognizable, while it was possible to discriminate between the two by UPLC‐ESI/MS analysis, emphasizing the high relevance of a precise true protein and chitin quantification for insects. To further confirm this hypothesis, chitin solubilization was measured for the digested BSF and for D‐BSF, at both digestion endpoints (ruminal and intestinal). As the chitin content in D‐BSF was below the instrumental limit of detection (LOD), the quantity of chitin of this sample was estimated based on the amount of BSF larvae present in the formulated diet and the percentage of chitin in the larvae (Table 1). The results are shown in Table 6.

**Table 6:** % Solubilized chitin after ruminal and intestinal digestion of black soldier fly larvae meal (BSF) and of black soldier fly larvae diet (D‐BSF).

Sample	Ruminal solubilization	Intestinal solubilization
BSF	25.3 ± 5.66	51.1 ± 0.55
D‐BSF	9.3 ± 2.28	48.3 ± 10.58

*Note:* The values are the average of two replicates ± SD.

Interestingly, the percentage of solubilized chitin after ruminal digestion was higher in the individually digested BSF than in D‐BSF. This might corroborate the previously mentioned discrepancy between higher CPD and lower AA solubilization of individually digested BSF larvae at ruminal endpoint, pointing out once again the determination of nitrogen origin and nature as a critical point in insect analysis. After abomasal and intestinal phases, however, differences in values between samples were evened out, being chitin solubilization around 50% for both BSF and D‐BSF.

Studies in the literature showed that chitinolytic microorganisms might be present in the rumen, thus indicating the possibility for chitin to be fermented and digested by ruminants (Fukuda et al. 2022). On the other hand, chitin and its deacetylated derivative, chitosan, might be able to modulate and lower CH_4_ production, which would be of extreme interest to decrease the environmental impact of the dairy cattle sector (Ahmed et al. 2021).

Most notably, our results indicate the presence of chitinolytic microorganisms in the collected rumen pool, as evidenced by chitin solubilization reaching up to 25.3% during this phase, corroborating the studies in the literature. The increase in chitin solubilization from ruminal to intestinal endpoint might imply a synergistic mechanism between microbial and chemical degradation: while the presence of solubilized chitin after the ruminal phase confirms the presence of active chitinolytic microorganisms capable of breaking the resilient β‐(1 → 4) glycosidic bonds of chitin, the subsequent exposure to highly acidic abomasal environment and intestinal enzymes effectively destabilized the structural bonds of the remaining chitin. Furthermore, this process likely helped increase protein solubilization after the intestinal phase: chitin breakdown might have exposed an otherwise undigestible fraction of cuticle proteins, which are usually closely linked to chitin, thus enabling enzymes to fully break down protein−chitin complexes. This led to approximately 90% protein solubilization, yielding protein‐free chitin and thereby enhancing both its hydrolysis and solubilization. In fact, after abomasal and intestinal digestion, differences in values between samples were evened out, chitin solubilization being around 50% for both BSF and D‐BSF.

### 3.3 Protein Integrity After Digestion: SDS‐PAGE and DH%

The MW distribution of proteins was determined by SDS‐PAGE analysis of D‐S, D‐N, D‐BSF and BSF, and on the filtered solutions derived from their digestion at both ruminal and intestinal endpoints. The electrophoretic profiles are shown in Figure 1.

**Figure 1:**
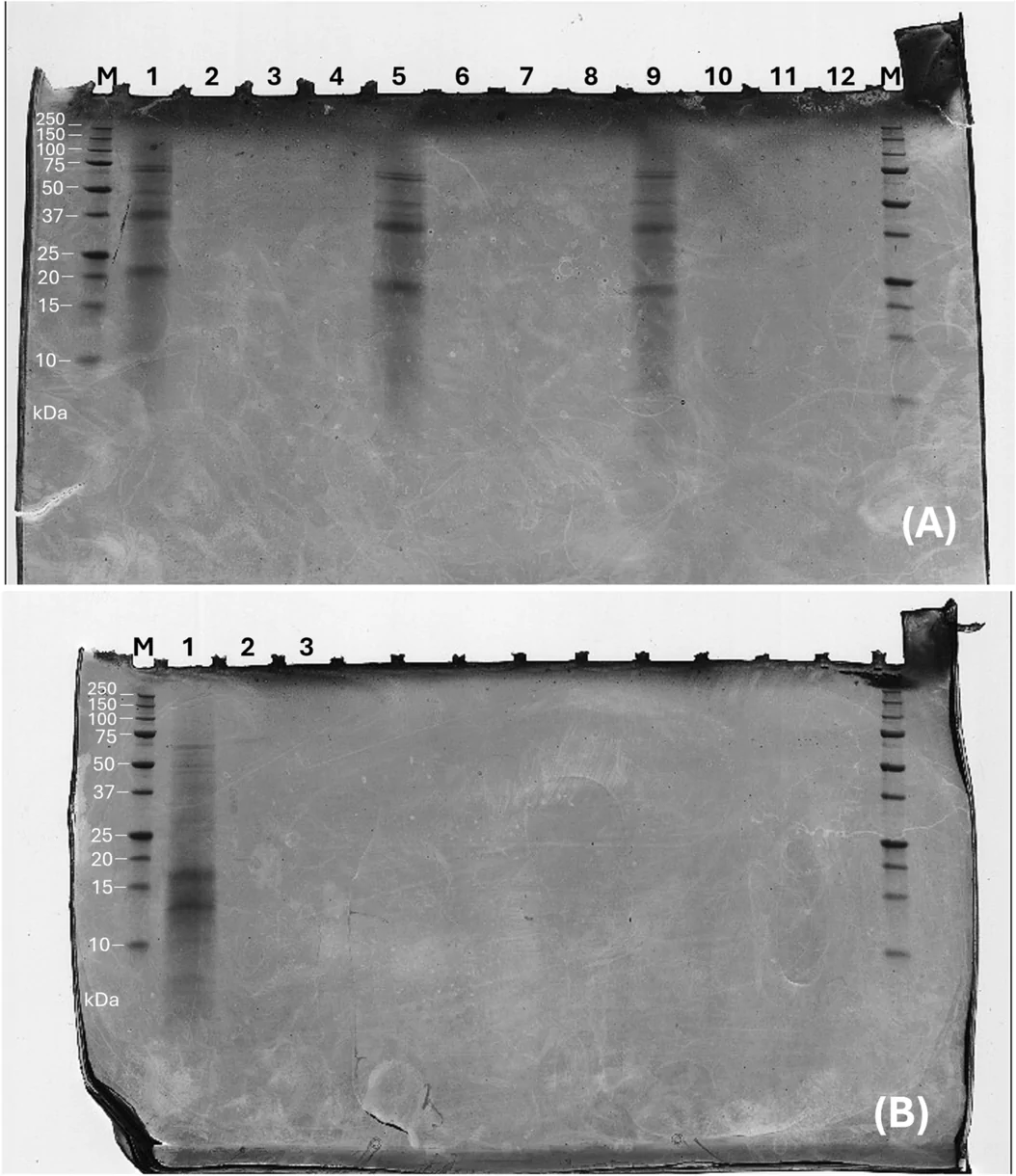
SDS‐PAGE analysis of undigested diets (D‐S: soybean diet; D‐N: protein feedstuff mix diet; D‐BSF: black soldier fly larvae diet), undigested black soldier fly whole larvae meal (BSF), and supernatants derived from their ruminal and intestinal digestion. (A) 1: D‐S undigested, 2: D‐S ruminal, 3: D‐S intestinal, 5: D‐N undigested, 6: D‐N ruminal, 7: D‐N intestinal, 9: D‐BSF undigested, 10: D‐BSF ruminal, 11: D‐BSF intestinal. (B) 1: BSF undigested, 2: BSF ruminal, 3: BSF intestinal.

D‐S, D‐N and D‐BSF in lanes 1, 5 and 9 of panel A, respectively, had a comparable protein profile, all showing the same defined protein bands around 75, 50, 37 and 20 kDa, thus meaning that the MW distribution of the three diets remained unchanged despite the different protein sources used, in line with the results on CPD. The BSF profile was comparable to that of previous works on the same matrix, with protein bands ranging from less than 10−75 kDa, the most prominent ones being around 20 kDa, attributable to cuticle proteins (Bonomini et al. 2024). Regarding the ruminal and intestinal filtered digesta solutions, no residual protein bands were detected for any of the samples. This means that the solubilized proteins after both digestion endpoints had a MW lower than 10 kDa, undetectable with the percentage of acrylamide used in this analysis, indicating that the ruminal microbiota in the first digestion phase (ruminal endpoint) and the combination of microorganisms and enzymes in the second phase (intestinal endpoint) were able to use and hydrolyse proteins to low MW. To further explore protein hydrolysis, the DH% was also assessed for all samples and for both digestion endpoints. The results are shown in Figure 2.

**Figure 2:**
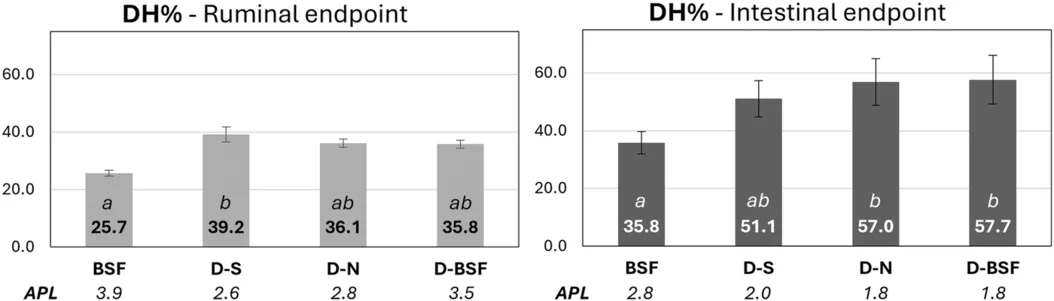
Degree of hydrolysis (DH%) and average peptide length (APL) after ruminal and intestinal digestion of the three formulated diets (D‐S: soybean diet; D‐N: protein feedstuff mix‐based diet; D‐BSF: black soldier fly larvae diet) and of black soldier fly whole larvae meal (BSF). The values are the average of three replicates ± SD. Different letters within the same endpoint refer to statistically significant differences between samples (one‐way ANOVA, Tukey's *b* post hoc test, *α* = 0.05).

Regarding the three diets, consistently with the expected progression of protein hydrolysis during the digestion process, the results indicated a higher DH% at intestinal endpoint, reaching 51.1%, 57.0% and 57.7% for D‐S, D‐N and D‐BSF, respectively, and the latter two were numerically higher than D‐S, although the differences were not significant. After the rumen, in fact, the abomasal and intestinal proteases (pepsin, trypsin, chymotrypsin) further hydrolyse proteins, cleaving a broader range of peptide bonds, leading to a higher DH%. This highlights the complementary nature of microbial and enzymatic proteolysis in ruminants. The APL decreased as the digestion phase advanced, with ruminal peptides being slightly longer (2.6–3.5) than intestinal peptides (1.8–2.0). The reduction in APL further corroborates the efficiency of abomasal and intestinal enzymes in breaking down oligopeptides into dipeptides and free AAs, and reflects the results on DH%, highlighting once more the efficiency of ruminants' multi‐phase digestion in breaking down dietary proteins. The DH% values for both ruminal and intestinal endpoints of the three diets were similar among samples, meaning that, despite the different protein sources with which the diets were formulated, protein hydrolysis remained overall unchanged, in agreement with previous results on protein digestibility of the samples under study.

Combining these results with protein solubilization after digestion, ruminal digestion results suggest that, while a majority of dietary proteins were solubilized (around 70%), the DH% was still relatively modest (35%–40%), indicating that microbial fermentation primarily produced oligopeptides, also aligning with the observed longer APL. Instead, the near‐complete protein solubilization (around 95%) and higher DH% (50%–60%) reflected the extensive protein breakdown, also in agreement with the observed lower APL (< 2).

### 3.4 Analysis of Insect Peptides Released After Ruminal and Intestinal Digestion

The filtered digesta solutions obtained from BSF and D‐BSF after both digestion endpoints (ruminal and intestinal) were analysed by a VIon IMS QTof mass spectrometer following the method, data processing and data filtering previously described, to further investigate insect protein digestibility. A summary of data is reported in Table 7, while the complete list of peptides detected is provided in Supporting Information S1: Table S2.

**Table 7:** Summary of descriptive data for black soldier fly whole larvae meal BSF (black soldier fly larvae) and black soldier fly larvae diet (D‐BSF) after high‐resolution mass spectrometric analysis of ruminal and intestinal digesta.

	BSF	D‐BSF
Ruminal digesta		
Total peptides detected	/	/
Total proteins detected	/	/
Intestinal digesta		
Total peptides detected	40	18
Total proteins detected	8	4
Medium peptide length	7.7	7.4

Surprisingly, no peptides were detected after the ruminal phase: considering that peptide fragments counting with less than three AA residues were excluded a priori during data processing but protein solubilization was detected, this result might actually mean that ruminal proteolytic activity mostly yielded free AA and small dipeptides, which is reasonable considering that the ruminal microorganisms carry out microbial protein synthesis, thus needing to break down the RDP into protein building blocks, AAs (Zain et al. 2024). This suggests that the peptides released during ruminal digestion are used and broken down by ruminal microbial populations and efficiently incorporated within microbial cells.

Regarding the intestinal endpoint, the D‐BSF showed a lower number of peptides detected (18) compared to BSF (40). Given the previously reported data on the DH%, which was higher in D‐BSF compared to BSF, this might mean that the protein solubilized were more extensively broken down in the formulated diet, thus yielding free AAs or small dipeptides which were undetected by the high‐resolution mass spectrometric analysis. Most of the peptides detected were derived from proteins with cuticular function or, if uncharacterized, showed high putative similarity with exoskeletal proteins (larval cuticular proteins, pupal cuticular proteins, flexible cuticular proteins, etc.) according to the Basic Local Alignment Search Tool (BLAST) on UniProt KB (Bonomini et al. 2024). As these proteins are usually tightly bound to chitin, which normally hinders their detachment and hydrolysis, the predominance of peptides from cuticular proteins in the intestinal digesta suggests that bovine digestion is able to metabolize chitin‐bound proteins, in agreement with the almost complete protein solubilization of both BSF and D‐BSF after intestinal digestion.

The medium peptide length, similar in BSF and D‐BSF, was in disagreement with the one calculated from the DH% of solubilized proteins, which was remarkably lower. Again, as peptides with less than three AA residues were not considered, while the DH% also takes into consideration free amino groups and dipeptides released after digestion, small protein fragments were not considered, thus resulting in an increase in the measured peptide length value.

## 4 Conclusions

The in vitro bovine digestibility of diets formulated with soybean (D‐S), with a commercial protein mix (D‐N) and with black soldier fly larvae (D‐BSF), and the digestibility of whole BSF meal as a single ingredient were assessed. To the best of our knowledge, this is the first study where the solubilization of individual AAs and chitin, DH%, MW distribution of proteins via SDS‐PAGE gel electrophoresis and HR‐MS analysis of peptides released after digestion were determined for a bovine in vitro trial. The study successfully demonstrated that whole BSF larvae could replace soybean meal in iso‐nitrogenous and iso‐energetic lactating dairy cattle diets without significantly compromising DMD, CPD, individual AA solubilization or DH%, despite the different intrinsic properties of the protein sources used to formulate the diets. Interestingly, chitin was solubilized up to approximately 50% in both D‐BSF and BSF: this supports the hypothesis that chitinolytic microorganisms are present in the rumen, and it confirms that ruminant digestive systems might effectively mitigate the anti‐nutritional constraints of chitin typically observed in monogastric species. These findings suggest that insects may be particularly advantageous as a feed ingredient for ruminants in comparison to monogastric species, as protein digestibility was unaffected by the presence of chitin, and as the partial solubilization of chitin itself would add nutritional value to the insect. To fully bridge the gap between laboratory and commercial applications, future research must prioritize long‐term in vivo feeding trials to evaluate the downstream impact of insect‐based feed on dairy product quality (composition and organoleptic profiles of milk, cheese, meat, etc.). Finally, addressing the current European regulatory barriers will require exhaustive risk assessments targeted at confirming the absence of transmissible spongiform encephalopathy vectors, which constitute the main concern around using insects as feed in ruminant species.

## Author Contributions

**Maria Giulia Bonomini:** conceptualization, data curation, formal analysis, investigation, writing – original draft. **Marica Simoni:** conceptualization, formal analysis, writing – review and editing. **Matteo Profeti:** formal analysis. **Federico Righi:** conceptualization, supervision, writing – review and editing. **Augusta Caligiani:** conceptualization, supervision, writing – review and editing.

## Conflicts of Interest

The authors declare no conflicts of interest.

## Supporting information


**Table S1:** Amino acid profile of the three diets (D‐S: soybean diet; D‐N: protein feedstuff mix diet; D‐BSF: black soldier fly larvae diet) and of black soldier fly whole larvae meal (BSF). The data are the average of three replicates ± SD. The results are expressed as g AA/100 g sample on a dry matter basis (DM).
**Table S2:** Peptides detected at intestinal endpoint after in vitro bovine digestion of BSF (black soldier fly larvae) and of D‐BSF (black soldier fly larvae diet).

## Data Availability

The data that support the findings of this study are available from the corresponding author upon reasonable request.

## References

[jpn70098-bib-0001] Accardo, F. , G. Leni , T. Tedeschi , B. Prandi , and S. Sforza . 2022. “Structural and Chemical Changes Induced by Temperature and pH Hinder the Digestibility of Whey Proteins.” Food Chemistry 387: 132884. 10.1016/j.foodchem.2022.132884. 35397269

[jpn70098-bib-0002] Ahmed, E. , N. Fukuma , M. Hanada , and T. Nishida . 2021. “Insects as Novel Ruminant Feed and a Potential Mitigation Strategy for Methane Emissions.” Animals: An Open Access Journal From MDPI 11, no. 9: 2648. 10.3390/ani11092648. PMC8471967 34573617

[jpn70098-bib-0003] Auza, F. A. , S. Purwanti , J. A. Syamsu , and A. Natsir . 2020. “Antibacterial Activities of Black Soldier Flies (*Hermetia illucens*. l) Extract Towards the Growth of *Salmonella typhimurium*, *E. coli* and *Pseudomonas aeruginosa* .” IOP Conference Series: Earth and Environmental Science 492, no. 1: 012024. 10.1088/1755-1315/492/1/012024.

[jpn70098-bib-0004] Bonomini, M. G. , B. Prandi , and A. Caligiani . 2024. “Black Soldier Fly (*Hermetia illucens* L.) Whole and Fractionated Larvae: In Vitro Protein Digestibility and Effect of Lipid and Chitin Removal.” Food Research International 196: 115102. 10.1016/j.foodres.2024.115102. 39614512

[jpn70098-bib-0005] Choi, W. H. , J. H. Yun , J. P. Chu , and K. B. Chu . 2012. “Antibacterial Effect of Extracts of *Hermetia illucens* (Diptera: Stratiomyidae) Larvae Against Gram‐Negative Bacteria.” Entomological Research 42, no. 5: 219–226. 10.1111/j.1748-5967.2012.00465.x.

[jpn70098-bib-0006] Churchward‐Venne, T. A. , P. J. M. Pinckaers , J. J. A. van Loon , and L. J. C. van Loon . 2017. “Consideration of Insects as a Source of Dietary Protein for Human Consumption.” Nutrition Reviews 75, no. 12: 1035–1045. 10.1093/nutrit/nux057. 29202184

[jpn70098-bib-0007] Dong, L. , R. M. C. Ariëns , A. H. P. America , et al. 2021. “ *Clostridium perfringens* Suppressing Activity in Black Soldier Fly Protein Preparations.” LWT 149: 111806. 10.1016/j.lwt.2021.111806.

[jpn70098-bib-0008] Estes, K. A. , P. S. Yoder , C. M. Stoffel , and M. D. Hanigan . 2022. “An Evaluation of the Validity of an In Vitro and an In Situ/In Vitro Procedure for Assessing Protein Digestibility of Blood Meal, Feather Meal and a Rumen‐Protected Lysine Prototype.” Translational Animal Science 6, no. 2: txac039. 10.1093/tas/txac039. PMC9071083 35529041

[jpn70098-bib-0009] European Commission (EC) . 2001. “Regulation (EC) No 999/2001 Laying Down Rules for the Prevention, Control and Eradication of Certain Transmissible Spongiform Encephalopathies.”

[jpn70098-bib-0010] European Commission (EC) . 2009. “Commission Regulation (EC) No 152/2009 of 27 January 2009 Laying Down the Methods of Sampling and Analysis for the Official Control of Feed.”

[jpn70098-bib-0011] European Commission (EC) . 2011. “Commission Implementing Regulation (EU) No 584/2011 of 17 June 2011 Approving Non‐Minor Amendments to the Specification for a Name Entered in the Register of Protected Designations of Origin and Protected Geographical Indications (Grana Padano (PDO)).”

[jpn70098-bib-0012] European Parliament . 2024. “Regulation (EU) 2024/1143 of the European Parliament and of the Council of 11 April 2024 on Geographical Indications for Wine, Spirit Drinks and Agricultural Products, as well as Traditional Specialities Guaranteed and Optional Quality Terms for Agricultural Products, Amending Regulations (EU) No 1308/2013, (EU) 2019/787 and (EU) 2019/1753 and Repealing Regulation (EU) No 1151/2012.”

[jpn70098-bib-0013] FAO . 2025. “FAOSTAT Database: Crops and Livestock Products.” https://www.fao.org/faostat/en/#data/QCL.

[jpn70098-bib-0014] Fukuda, E. P. , J. R. Cox , T. A. Wickersham , and M. L. Drewery . 2022. “Evaluation of Black Soldier Fly Larvae (*Hermetia illucens*) as a Protein Supplement for Beef Steers Consuming Low‐Quality Forage.” Translational Animal Science 6, no. 1: txac018. 10.1093/tas/txac018. PMC8882252 35233513

[jpn70098-bib-0045] Goering, H. K. , and P. J. Van Soest . 1970. “Forage Fiber Analysis (Apparatus, Reagents, Procedures and some Applications).” In Agriculture Handbook 379. U.S. Agricultural Research Service.

[jpn70098-bib-0015] Harlystiarini, H. , R. Mutia , I. W. T. Wibawan , and D. A. Astuti . 2019. “In Vitro Antibacterial Activity of Black Soldier Fly (*Hermetia illucens*) Larva Extracts Against Gram‐Negative Bacteria.” Buletin Peternakan 43, no. 2: 125–129. 10.21059/buletinpeternak.v43i2.42833.

[jpn70098-bib-0016] Hawkey, K. J. , C. Lopez‐Viso , J. M. Brameld , T. Parr , and A. M. Salter . 2021. “Insects: A Potential Source of Protein and Other Nutrients for Feed and Food.” 10.1146/annurev-animal-021419. 33228376

[jpn70098-bib-0017] Hristov, A. N. , A. Bannink , L. A. Crompton , et al. 2019. “Invited Review: Nitrogen in Ruminant Nutrition: A Review of Measurement Techniques.” Journal of Dairy Science 102, no. 7: 5811–5852. 10.3168/jds.2018-15829. 31030912

[jpn70098-bib-0018] Ismail, B. P. , L. Senaratne‐Lenagala , A. Stube , and A. Brackenridge . 2020. “Protein Demand: Review of Plant and Animal Proteins Used in Alternative Protein Product Development and Production.” Animal Frontiers 10, no. 4: 53–63. 10.1093/af/vfaa040. PMC7759735 33391860

[jpn70098-bib-0019] Janssen, R. H. , J. P. Vincken , L. A. M. Van Den Broek , V. Fogliano , and C. M. M. Lakemond . 2017. “Nitrogen‐to‐Protein Conversion Factors for Three Edible Insects: *Tenebrio molitor*, *Alphitobius diaperinus*, and *Hermetia illucens* .” Journal of Agricultural and Food Chemistry 65, no. 11: 2275–2278. 10.1021/acs.jafc.7b00471. PMC5364430 28252948

[jpn70098-bib-0020] Jayanegara, A. , B. Novandri , N. Yantina , and M. Ridla . 2017. “Use of Black Soldier Fly Larvae (*Hermetia illucens*) to Substitute Soybean Meal in Ruminant Diet: An In Vitro Rumen Fermentation Study.” Veterinary World 10, no. 12: 1436–1446. 10.14202/vetworld.2017.1439-1446. PMC5771168 29391684

[jpn70098-bib-0021] Kumar, D. , C. Datt , L. K. Das , and S. S. Kundu . 2015. “Evaluation of Various Feedstuffs of Ruminants in Terms of Chemical Composition and Metabolisable Energy Content.” Veterinary World 8, no. 5: 605–609. 10.14202/vetworld.2015.605-609. PMC4774720 27047142

[jpn70098-bib-0023] Lu, S. , N. Taethaisong , W. Meethip , et al. 2022. “Nutritional Composition of Black Soldier Fly Larvae (*Hermetia illucens* L.) and Its Potential Uses as Alternative Protein Sources in Animal Diets: A Review.” In Insects. MDPI. 10.3390/insects13090831. PMC9502457 36135532

[jpn70098-bib-0022] Lu, S. , S. Chen , S. Paengkoum , et al. 2024. “Effects of Black Soldier Fly (*Hermetia illucens* L., BSF) Larvae Addition on In Vitro Fermentation Parameters of Goat Diets.” Insects 15, no. 5: 343. 10.3390/insects15050343. PMC11121845 38786899

[jpn70098-bib-0024] Maggitt, S. , J. Cox , K. Dobson , J. McCann , T. A. Wickersham , and M. L. Drewery . 2025. “Intake, Digestion, and Rumen Microbial Impacts of Black Soldier Fly Larvae and Frass Provided as Protein Supplements to Cattle Consuming Forage.” Journal of Animal Science 103: skaf077. 10.1093/jas/skaf077. PMC12065404 40065635

[jpn70098-bib-0042] Mertens, D. R . 2002. Measuring Fiber and Its Effectiveness in Ruminant Diets. https://api.semanticscholar.org/CorpusID:3223462.

[jpn70098-bib-0044] Mihaljev, Ž. A. , S. M. Jakšić , N. B. Prica , Ž. N. Ćupić , and M. M. Živkov‐Baloš . 2015. “Comparison of the Kjeldahl Method, Dumas Method and NIR Method for Total Nitrogen Determination in Meat and Meat Products.” Journal of Agroalimentary Processes and Technologies 21, no. 4: 365–370.

[jpn70098-bib-0025] NASEM (National Academies of Sciences, Engineering, and Medicine) . 2021. Nutrient Requirements of Dairy Cattle. 8th ed. National Academies Press. 10.17226/25806. 38386771

[jpn70098-bib-0026] Nekrasov, R. V. , G. A. Ivanov , M. G. Chabaev , et al. 2022. “Effect of Black Soldier Fly (*Hermetia illucens* L.) Fat on Health and Productivity Performance of Dairy Cows.” Animals: An Open Access Journal From MDPI 12, no. 16: 2118. 10.3390/ani12162118. PMC9405003 36009708

[jpn70098-bib-0027] Paz, H. A. , T. J. Klopfenstein , D. Hostetler , S. C. Fernando , E. Castillo‐Lopez , and P. J. Kononoff . 2014. “Ruminal Degradation and Intestinal Digestibility of Protein and Amino Acids in High‐Protein Feedstuffs Commonly Used in Dairy Diets.” Journal of Dairy Science 97, no. 10: 6485–6498. 10.3168/jds.2014-8108. 25108871

[jpn70098-bib-0028] Pedrazzani, C. , L. Righi , F. Vescovi , L. Maistrello , and A. Caligiani . 2024. “Black Soldier Fly as a New Chitin Source: Extraction, Purification and Molecular/Structural Characterization.” LWT 191: 115618. 10.1016/j.lwt.2023.115618.

[jpn70098-bib-0029] Refael, G. , H. T. Riess , C. S. Levi , et al. 2022. “Responses of the Human Gut Microbiota to Physiologically Digested Insect Powders or Isolated Chitin Thereof.” Future Foods 6: 100197. 10.1016/j.fufo.2022.100197.

[jpn70098-bib-0030] Renna, M. , L. Rastello , T. Veldkamp , et al. 2023. “Are Insects a Solution for Feeding Ruminants? Legislation, Scientific Evidence, and Future Challenges.” Animal Frontiers 13, no. 4: 102–111. 10.1093/af/vfad026. PMC10425146 37583801

[jpn70098-bib-0031] Ritchie, H. , P. Rosado , and M. Roser . 2022. “Environmental Impacts of Food Production.” Our World in Data. https://ourworldindata.org/environmental-impacts-of-food.

[jpn70098-bib-0043] Robertson, J. B. , and P. J. Van Soest . 1981. “The Detergent System of Analysis and its Application to Human Foods.” In The Analysis of Dietary Fiber in Food, 123–158. Marcel Dekker.

[jpn70098-bib-0032] Ross, D. A. , M. Gutierrez‐Botero , and M. E. Van Amburgh . 2013. “Development of an In Vitro Intestinal Digestibility Assay for Ruminant Feeds.” http://www.patentstorm.us/patents/5422108/description.html;.

[jpn70098-bib-0033] Shah, A. M. , I. H. Qazi , M. Matra , and M. Wanapat . 2022. “Role of Chitin and Chitosan in Ruminant Diets and Their Impact on Digestibility, Microbiota and Performance of Ruminants.” Fermentation 8, no. 10: 549. 10.3390/fermentation8100549.

[jpn70098-bib-0035] Simoni, M. , R. Temmar , M. De Marchi , et al. 2024. “Milking System and Diet Forage Type Effect on Milk Quality of Italian Holstein‐Friesian.” Journal of Dairy Science 107, no. 9: 6983–6993. 10.3168/jds.2023-24464. 38825097

[jpn70098-bib-0034] Simoni, M. , T. Danese , A. Guerra , et al. 2024. “Evaluating NIRS for Predicting Faecal Fibre, Protein Fractions and Digestibility in Dairy Sheep and Goats Fed Alfalfa Hay and Concentrate.” Italian Journal of Animal Science 23, no. 1: 1479–1490. 10.1080/1828051X.2024.2409967.

[jpn70098-bib-0036] Sogari, G. , S. Bellezza Oddon , L. Gasco , A. van Huis , T. Spranghers , and S. Mancini . 2023. “Review: Recent Advances in Insect‐Based Feeds: From Animal Farming to the Acceptance of Consumers and Stakeholders.” In Animal, 100904. Elsevier B.V. 10.1016/j.animal.2023.100904. 37500376

[jpn70098-bib-0037] Spranghers, T. , J. Michiels , J. Vrancx , et al. 2018. “Gut Antimicrobial Effects and Nutritional Value of Black Soldier Fly (*Hermetia illucens* L.) Prepupae for Weaned Piglets.” Animal Feed Science and Technology 235: 33–42. 10.1016/j.anifeedsci.2017.08.012.

[jpn70098-bib-0038] Toral, P. G. , G. Hervás , M. G. González‐Rosales , A. G. Mendoza , L. E. Robles‐Jiménez , and P. Frutos . 2022. “Insects as Alternative Feed for Ruminants: Comparison of Protein Evaluation Methods.” Journal of Animal Science and Biotechnology 13, no. 1: 21. 10.1186/s40104-021-00671-2. PMC8832790 35144685

[jpn70098-bib-0039] van Huis, A. , and L. Gasco . 2023. “Insects as Feed for Livestock Production.” Science 379, no. 6628: 138–139. 10.1126/science.adc9165. 36634163

[jpn70098-bib-0040] Van Moll, L. , J. De Smet , A. Paas , et al. 2022. “In Vitro Evaluation of Antimicrobial Peptides from the Black Soldier Fly (*Hermetia illucens*) Against a Selection of Human Pathogens.” https://journals.asm.org/journal/spectrum. 10.1128/spectrum.01664-21 PMC8729770 34985302

[jpn70098-bib-0041] Zain, M. , U. H. Tanuwiria , J. A. Syamsu , et al. 2024. “Nutrient Digestibility, Characteristics of Rumen Fermentation, and Microbial Protein Synthesis From Pesisir Cattle Diet Containing Non‐Fiber Carbohydrate to Rumen Degradable Protein Ratio and Sulfur Supplement.” Veterinary World 17, no. 3: 672–681. 10.14202/vetworld.2024.672-681. PMC11045530 38680159

