# Supplementary material for: Evaluating the In Vitro Digestibility of Black Soldier Fly (Hermetia illucens L.) Larvae as Potential Feed Ingredient in Dairy Cattle Silage‐Based Diets: A Focus on Proteins
Source: J Anim Physiol Anim Nutr (Berl). 2026 Jul 30;110(6):849–60. doi: 10.1111/jpn.70098 (PMC13768946; doi:10.1111/jpn.70098)
Supplement: Supplementary file 1 [file JPN-110-849-s001.docx]

**Supplementary material**

**Supplementary Table S1**. Amino acid profile of the three diets (D-S: soybean diet; D-N: protein feedstuff mix diet; D-BSF: black soldier fly larvae diet) and of black soldier fly whole larvae meal (BSF). The data are the average of three replicates ± SD. The results are expressed as g AA/100 g sample on a dry matter basis (DM).

| Amino acid | **D-S** | **D-N** | **D-BSF** | **BSF** |
| --- | --- | --- | --- | --- |
| Ala | 2.20±0.10 | 1.97±0.05 | 2.18±0.09 | 3.87±0.02 |
| Arg | 0.98±0.08 | 0.86±0.07 | 0.86±0.06 | 1.70±0.02 |
| Asp/Asn | 3.04±0.13 | 2.62±0.17 | 2.65±0.03 | 4.57±0.12 |
| Glu/Gln | 4.69±0.24 | 4.21±0.16 | 4.25±0.26 | 6.23±0.13 |
| Gly | 1.57±0.06 | 1.45±0.04 | 1.51±0.09 | 2.75±0.02 |
| His | 0.58±0.01 | 0.54±0.02 | 0.56±0.05 | 0.86±0.01 |
| Ile | 1.28±0.12 | 1.11±0.06 | 1.12±0.03 | 2.03±0.01 |
| Leu | 2.93±0.21 | 2.57±0.10 | 2.55±0.11 | 3.88±0.05 |
| Lys | 0.21±0.03 | 0.16±0.01 | 0.21±0.02 | 0.85±0.12 |
| Phe | 1.76±0.10 | 1.58±0.07 | 1.54±0.04 | 2.20±0.07 |
| Pro | 2.44±0.26 | 2.09±0.04 | 2.30±0.35 | 3.27±0.26 |
| Ser | 1.62±0.10 | 1.47±0.05 | 1.52±0.05 | 2.38±0.06 |
| Thr | 1.36±0.09 | 1.24±0.04 | 1.27±0.06 | 2.17±0.02 |
| Tyr | 1.09±0.05 | 0.98±0.04 | 1.10±0.06 | 2.95±0.10 |
| Val | 1.65±0.10 | 1.44±0.08 | 1.50±0.07 | 3.08±0.05 |
| **^†^Total amino acids** | 27.4±0.13 | 24.3±0.08 | 25.1±0.13 | 42.8±0.10 |
| **^‡^True protein** | 23.4±0.11 | 20.7±0.07 | 21.4±0.11 | 36.5±0.08 |

^†^Calculated with the hydrated molecular weight

^‡^Calculated with the residual molecular weight

**Supplementary Table S2**. Peptides detected at intestinal endpoint after *in vitro* bovine digestion of BSF (black soldier fly larvae) and of D-BSF (black soldier fly larvae diet)

| **A0A7R8ULW8** | Uncharacterized protein | | | |
| --- | --- | --- | --- | --- |
| Position | Peptide | Length | BSF | D-BSF |
| 51-58 | VSPEGTPI | 8 |  |  |
|  |  |  |  |  |
| **A0A7R8UMF6** | Uncharacterized protein | | | |
| Position | Peptide | Length | BSF | D-BSF |
| 13-24 | RLEADNIGTDGY | 12 |  |  |
|  |  |  |  |  |
| **A0A7R8UNL5** | Larval cuticle protein A2B | | | |
| Position | Peptide | Length | BSF | D-BSF |
| 13-21 | PVVAAAAPV | 9 |  |  |
| 172-177 | ASPIVS | 6 |  |  |
| **A0A7R8URU7** | Uncharacterized protein | | | |
| Position | Peptide | Length | BSF | D-BSF |
| 73-80 | GAGIGSGL | 8 |  |  |
| 138-145 | GVGIPVPV | 8 |  |  |
| 138-146 | GVGIPVPVQ | 9 |  |  |
| 139-145 | VGIPVPV | 7 |  |  |
| 141-145 | IPVPV | 5 |  |  |
| 148-153 | NIPVPV | 6 |  |  |
| 162-169 | SVPVPQPY | 8 |  |  |
| 163-167 | VPVPQ | 5 |  |  |
| 163-169 | VPVPQPY | 7 |  |  |
| 198-204 | VPVAAVA | 7 |  |  |
| 206-213 | PVDRPYPV | 8 |  |  |
| 226-231 | TIAVPV | 6 |  |  |
| 271-279 | VQVDRPVPV | 9 |  |  |
| 273-279 | VDRPVPV | 7 |  |  |
| 274-279 | DRPVPV | 6 |  |  |
| 284-289 | PVPVPV | 6 |  |  |
| 288-295 | PVERPVPV | 8 |  |  |
| 307-315 | VNVDRPVPV | 9 |  |  |
| 308-315 | NVDRPVPV | 8 |  |  |
| 324-331 | PVQVPVPQ | 8 |  |  |
| 324-333 | PVQVPVPQPY | 10 |  |  |
| 334-341 | SVDRPVPV | 8 |  |  |
| 334-343 | SVDRPVPVPV | 10 |  |  |
| 335-342 | VDRPVPVP | 8 |  |  |
| 335-343 | VDRPVPVPV | 9 |  |  |
| 336-343 | DRPVPVPV | 8 |  |  |
| 376-383 | PVPVPSPV | 8 |  |  |
| 376-384 | PVPVPSPVS | 9 |  |  |
| 384-390 | SVGVGAI | 7 |  |  |
| 392-399 | LGATAAPL | 8 |  |  |
| 393-399 | GATAAPL | 7 |  |  |
| 460-467 | ASGALLGT | 8 |  |  |
|  |  |  |  |  |
| **A0A7R8USN4** | Ferritin | | | |
| Position | Peptide | Length | BSF | D-BSF |
| 107-111 | VTNLI | 5 |  |  |
|  |  |  |  |  |
| **A0A7R8YT67** | Larval cuticle protein A2B | | | |
| Position | Peptide | Length | BSF | D-BSF |
| 36-42 | LSVNAGL | 7 |  |  |
| 118-124 | VVAAAPV | 7 |  |  |
| 198-204 | VPVAAVA | 7 |  |  |
|  |  |  |  |  |
|  |  | | | |
| **A0A7R8Z063** | Uncharacterized protein | | | |
| Position | Peptide | Length | BSF | D-BSF |
| 53-60 | TSPEGIPI | 8 |  |  |
|  |  |  |  |  |
| **A0A7R8Z2D0** | Pupal cuticle protein Edg-78E | | | |
| Position | Peptide | Length | BSF | D-BSF |
| 98-104 | PQPPPIP | 7 |  |  |
